# Occipital GABA levels in older adults and their relationship to visual perceptual suppression

**DOI:** 10.1038/s41598-017-14577-5

**Published:** 2017-10-27

**Authors:** Kabilan Pitchaimuthu, Qi-zhu Wu, Olivia Carter, Bao N. Nguyen, Sinyeob Ahn, Gary F. Egan, Allison M. McKendrick

**Affiliations:** 1 Department of Optometry and Vision Sciences, University of Melbourne, Parkville, VIC 3010 Australia; 2 Monash Biomedical Imaging, Monash University, Clayton, VIC 3800 Australia; 3 Melbourne School of Psychological Sciences, University of Melbourne, Parkville, VIC 3010 Australia; 4 Magnetic Resonance, Siemens Medical Solutions USA Inc, San Francisco, CA 94116 USA

## Abstract

Several studies have attributed certain visual perceptual alterations in older adults to a likely decrease in GABA (Gamma Aminobutyric Acid) concentration in visual cortex, an assumption based on findings in aged non-human primates. However, to our knowledge, there is no direct evidence for an age-related decrease in GABA concentration in human visual cortex. Here, we estimated visual cortical GABA levels and Glx (combined estimate of glutamate and glutamine) levels using magnetic resonance spectroscopy. We also measured performance for two visual tasks that are hypothesised to be mediated, at least in part, by GABAergic inhibition: spatial suppression of motion and binocular rivalry. Our results show increased visual cortical GABA levels, and reduced Glx levels, in older adults. Perceptual performance differed between younger and older groups for both tasks. When subjects of all ages were combined, visual cortical GABA levels but not Glx levels correlated with perceptual performance. No relationship was found between perception and GABA levels in dorsolateral prefrontal cortex. Perceptual measures and GABA were not correlated when either age group was considered separately. Our results challenge current assumptions regarding neurobiological changes that occur within the aging human visual cortex and their association with certain age-related changes in visual perception.

## Introduction

GABAergic inhibition is central to the output and activity of visual neural circuitry^1–4^. There is a prevailing view that healthy ageing results in impaired GABAergic inhibition^5–11^, a view that has been motivated largely by observations from the visual system of older non-human primates^10^. Specifically, many neurones in the primary visual cortex (V1) of older macaques exhibit reduced orientation selectivity^10,11^ that is partly restored by the application of GABA and GABA_A_ agonist^10^. The authors noted that this observation is potentially consistent with possible reduced synthesis of GABA in the visual cortex of older monkeys, however, GABA synthesis was not directly tested. Changes to the GABA system in visual cortex are not isolated to primates, with subsequent work in cats showing that the proportion of GABAergic neurons to total neurones is reduced in V1^12^.

At a single cell level within the visual cortex, there are numerous experimental observations that are consistent with reduced GABAergic inhibition in older animals. For example, single cell studies demonstrate increased spontaneous activity^10,11,13^, decreased orientation and direction selectivity^10,11^, and reduced surround suppression^8^ in V1 of older primates. At a perceptual level in humans, however, a simple model of reduced GABA levels in visual cortex does not readily explain observations of unaltered levels of orientation discrimination^14,15^, increased centre surround contrast suppression^16–19^, and prolonged binocular rivalry percept durations^20,21^ in older adults compared to younger adults. Indeed, some of these findings are more consistent with increased intracortical inhibition^16–18,22^. However, there are other perceptual findings in older adults that are consistent with reduced inhibitory strength, in particular, the observation of reduced spatial suppression of motion discrimination^6,18,23^. None of these previous perceptual studies have measured cortical GABA concentration, but have discussed theoretically.

Magnetic resonance spectroscopy (MRS) has been shown to robustly estimate *in-vivo* GABA levels. Recent studies in younger adults^24–26^, and several patient groups^27,28^, have shown that such estimates of GABA concentration correlate with the strength of several perceptual effects that are considered to rely on inhibitory function, namely: surround contrast suppression^24,28^, orientation discrimination^29^, and binocular rivalry^26,27^. Consequently, GABA measurement by MRS has the potential to be a useful tool to explore whether changes to presumed perceptual inhibitory strength within the visual system can be explained, at least partially, by differences in GABA levels between individuals. Previous MRS studies have demonstrated reduced GABA levels in the right hippocampus^30^, frontal^31^ and parietal^31^ cortices in older adults, and unaltered levels of GABA concentration in anterior cingulate cortex of older adults^30,32^. However, there is an absence of measurements of visual cortical GABA concentration in people of varying age, and therefore a scarcity of data to assist in explaining some apparent contradictions between studies measuring age related inhibitory changes at physiological and perceptual levels.

Hence, here we measured GABA levels in the visual cortex of older and younger adults using magnetic resonance spectroscopy (MRS). We also assessed perceptual performance on the same day for two visual tasks that are considered to be mediated, at least in part, by GABAergic inhibition: spatial suppression of motion discrimination (spatial suppression, hereafter) and binocular rivalry. We aimed to determine whether the magnitude of perceptual inhibition correlates with measured GABA in human visual cortex. Intriguingly, increased GABA concentration was present in the older adult group. We discuss our findings in the context of models of visual inhibition of relevance to the specific perceptual tasks studied, and in the context of possible changes to cellular GABA and GABAergic neurotransmission that cannot be directly measured by MRS.

## Results

### Elevated GABA levels in visual cortex of older adults

**Figure 1:**
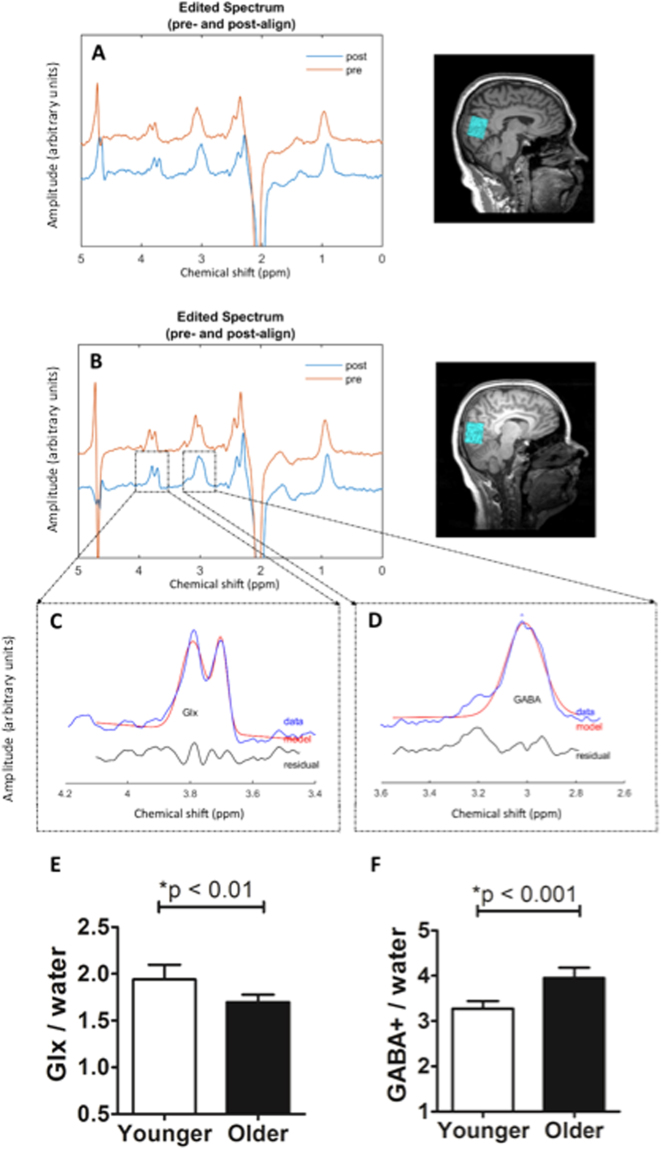
GABA and Glx quantification details and GABA+/water and Glx/water levels in younger and older adults. An example spectrum from the visual cortex of an older participant (**A**) and a younger participant (**B**) showing pre- (red) and post- (blue) frequency and phase correction by Gannet. Gannet fitting of 3.0 ppm GABA peak (**C**) and 3.75 ppm edited Glx peaks (**D**) from the spectrum shown on panel B; concentrations were quantified by the area under the curve. Group mean GABA+/water and Glx/water concentration in visual cortex of younger (**E**) and older adults (**F**). Error bars represent the 95% confidence intervals of the mean.

Visual cortical GABA levels were measured in 18 younger (mean 28; 20-34 years) and 20 older adults (mean 71; 63–78 years) using an occipital voxel. The 3 T MRS procedure (MAGNETOM Skyra, Siemens, with 32 channel head coil) was conducted using a GABA-specific MEGA PRESS sequence^33^ with GABA levels quantified from the j-edited difference spectrum using Gannet software^25^ (see Methods for details of the experimental procedure). Since the edited GABA signal is contaminated by co-edited macromolecules^32^, GABA values are referred to as GABA+ in this paper. GABA+ values were normalised to water and adjusted for the tissue composition of the voxel (for details of the group differences between tissue composition of the visual cortical voxel, see Supplementary Figure S1A–C). Example spectra are shown in Fig. 1 (A: older adult; B: younger adult; C and D: fitting of Glx and GABA+ peaks; E and F: group mean Glx/water and GABA+/water in young and older adults; spectra from all participants are included in Supplementary Figure S2). As shown in Fig. 1F, there was an approximate 21% increase in GABA+/water levels in the visual cortex of older adults (mean ± stdev = 3.95 ± 0.49), relative to younger adults (3.28 ± 0.33; t(1,36) = −4.92, P < 0.001). The effect size of this increase in visual cortical GABA+/water levels with ageing was large (Cohen’s *d* = 1.62). As this study was primarily aimed at identifying GABA related changes in the ageing cortex, the MEGA PRESS sequence used was optimised for the GABA signal. It was also possible to estimate the levels of edited Glx, a combined estimate of glutamate and glutamine. In contrast to the elevated GABA levels, Glx/water levels were reduced in older adults (Fig. 1E, mean ± stdev = 1.70 ± 0.17), compared to younger adults (1.94 ± 0.31; t(1,36) = 3.02, P < 0.01; large effect size: Cohen’s *d* = 0.97).

### Visual cortical GABA+/water levels correlate with visual function

**Figure 2:**
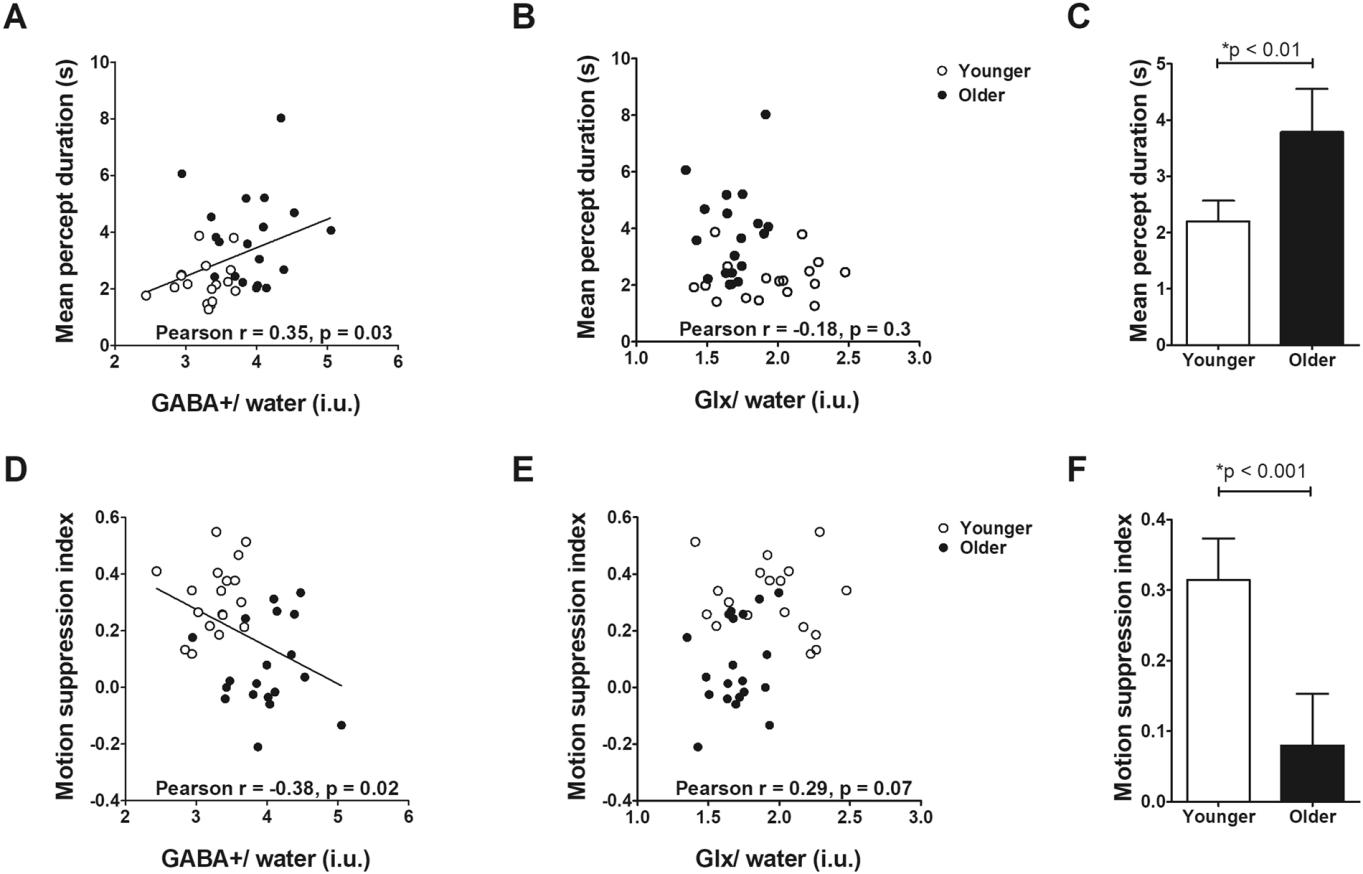
Correlation of visual task performance with visual cortical GABA+/water. (**A**) Mean percept duration on the binocular rivalry task was positively correlated with GABA+/water in visual cortex, but not with Glx/water (**B**). (**C**) Older adults (filled) showed longer mean percept duration than younger adults (unfilled) indicating increased interocular suppression in the binocular rivalry task. (**D**) Motion suppression index was negatively correlated with GABA+/water in visual cortex, and (**E**) a trend towards a positive correlation between motion suppression and Glx/water levels (**F**) Older adults (filled) showed reduced spatial suppression of motion (lower motion suppression indices) than younger adults (unfilled). Error bars represent 95% confidence intervals of the mean. Bonferroni correction would result in a p-value of 0.0125 being considered for statistical significance for the correlations.

Within one hour of the MRS procedure, we measured performance on two visual tasks where performance is possibly related to GABAergic inhibition: binocular rivalry and spatial suppression of motion. We measured binocular rivalry using two sine gratings projected to two eyes (right eye: red grating, oriented 45°, left eye: green grating, oriented 135°). Participants indicated when they observed a red percept, green percept or mixed percept by button press (for detailed procedures, see Methods). Results for the binocular rivalry task are shown in Fig. 2A–C. In line with previous literature in younger adults^26,27^, we found that increased GABA+/water levels were associated with longer percept durations (Fig. 2A, *Pearson* r = 0.35, p = 0.03) and decreased number of perceptual switches (*Pearson* r = 0.36, p = 0.03) when considered across the entire sample (older and younger adults). In addition, similar to the findings of van Loon *et al*.^26^, visual cortical Glx/water levels did not correlate with either mean percept duration (Fig. 2B, *Pearson* r = −0.18, p = 0.30) or switch rate (*Pearson* r = 0.25, p = 0.14). Moreover, older adults showed longer percept durations (Fig. 2C, *t*(1,34) = 3.67, P < 0.01) and reduced perceptual switches (*t*(1,34) = 4.03, P < 0.001) than younger adults, a finding consistent with previous behavioural studies^20,21,34^. When each age group was considered in isolation, there were no significant correlations between either MRS measure and percept duration.

We next determined the relationship between GABA+/water levels in visual cortex and the magnitude of perceptual spatial suppression. The task required participants to identify the left or right direction of motion of large, high-contrast (92%) drifting Gabor patterns. Paradoxically, observers require longer stimulus presentation durations as the stimulus size increases^35^, an observation that is considered to reflect the centre-surround antagonistic receptive field properties of MT/V5 neurones mediating human motion perception^35,36^. Here, we tested two sizes (2σ of Gaussian envelope: 0.7° and 5°) (further details in Methods). Suppression indices were calculated as follows:

1 $${\rm{Motion}}\,{\rm{suppression}}\,{\rm{index}}={\mathrm{log}}_{{\rm{10}}}({\rm{Duration}}\,{{\rm{Threshold}}}_{{\rm{5^\circ }}})\,-\,{\mathrm{log}}_{{\rm{10}}}({{\rm{DurationThreshold}}}_{{\rm{0.7^\circ }}}).$$

Higher GABA+/water levels in visual cortex were associated with reduced motion suppression indices when both age groups were considered (Fig. 2D, *Pearson* r = −0.38, P = 0.02). Visual cortical Glx/water levels did not correlate with spatial suppression, however there was a trend towards increased Glx/water levels associated with increased spatial suppression (Fig. 2E, *Pearson* r = 0.29, P = 0.07). In addition, older adults showed less spatial suppression of motion than younger adults (Fig. 2F, *t*(1, 36) = 5.15, P < 0.001), consistent with previous literature^6,18,23^.

### Regional specificity of findings

**Figure 3:**
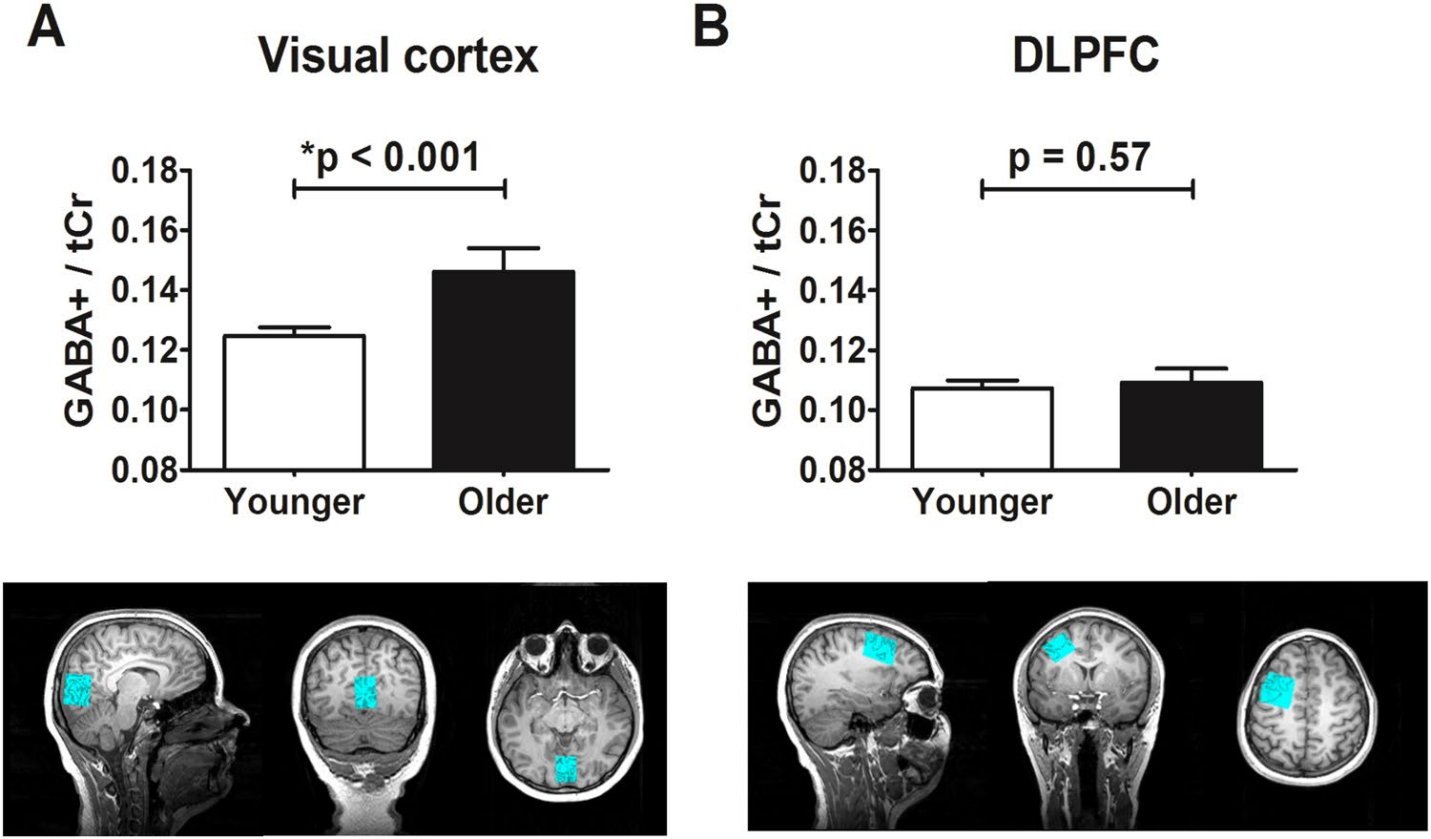
GABA+/tCr levels in visual cortex and in DLPFC. (**A**) GABA+/tCr levels in visual cortex were increased in older adults (filled) compared to younger adults (unfilled). (**B**) Both older (filled) and younger (unfilled) adults had similar amounts GABA+/tCr in DLPFC. Error bars represent 95% confidence intervals of the mean. Bonferroni correction would result in a p-value of 0.025 being considered for statistical significance.

To determine whether our findings were anatomically specific to visual cortex, we also placed a voxel of the same size as that used to measure from visual cortex in the dorsolateral prefrontal cortex (DLPFC). We did not collect unsuppressed water data in DLPFC so for this analysis we normalised GABA+ to total creatine (tCr) (see Methods for details). Figure 3 compares the levels of GABA+/tCr between younger and older adults in both visual cortex and DLPFC. Visual cortical GABA+/tCr levels were elevated in older adults (Fig. 3A, *t*(1,36) = −4.47, P < 0.001), however GABA+/tCr levels in DLPFC did not differ between younger and older adults (Fig. 3B, *t*(1,36) = −0.57, P = 0.57).

**Figure 4:**
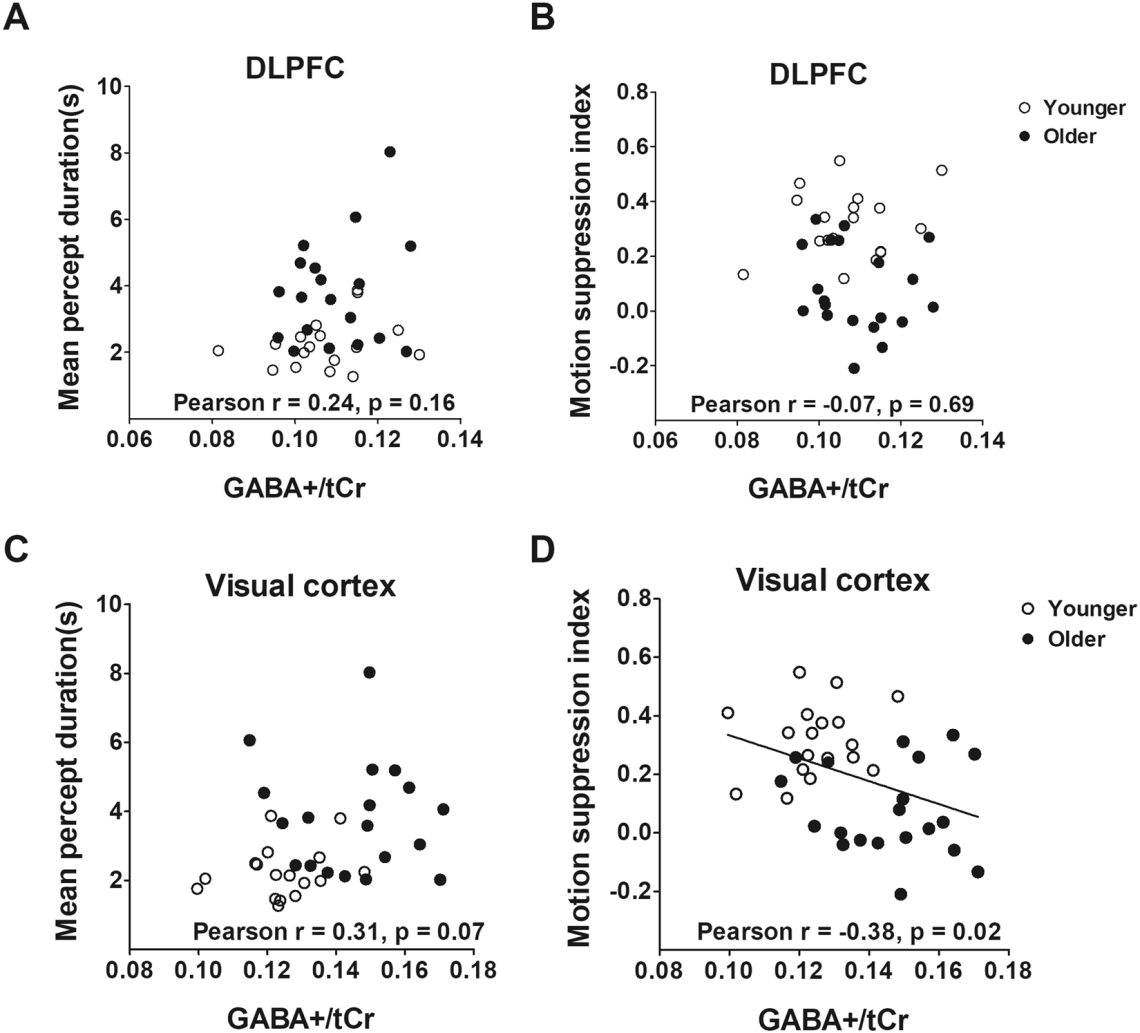
Correlation of visual task performances with GABA+/tCr levels in DLPFC and visual cortex. GABA+/tCr levels in DLPFC did not correlate with either binocular rivalry percept durations (**A**) or spatial suppression (**B**). GABA+/tCr levels in visual cortex had a trend towards positive correlation with binocular rivalry percept durations (**C**) and a negative correlation with spatial suppression (**D**). Bonferroni correction would result in a p-value of 0.0125 being considered for statistical significance.

Neither spatial suppression nor binocular rivalry performance correlated with GABA+/tCr levels measured in DLPFC (Fig. 4A *binocular rivalry: Pearson* r = 0.24, p = 0.16; Fig. 4B *motion suppression: Pearson* r = −0.07, p = 0.69). Figure 4C and D show the results for visual cortex where the correlation with binocular rivalry mean percept duration approached significance (Pearson r = 0.3, p = 0.07), whereas the correlation with spatial suppression reached conventional statistical significance (Pearson r = −0.38, p = 0.02). Note, our main visual cortical findings (Fig. 2) are referenced to water, as this is the most contemporary reference standard for such MRS work. We repeated the visual cortical analysis for GABA+/tCr here to demonstrate that the findings were not specific to the referencing technique (Fig. 4).

## Discussion

Using MRS, we demonstrate elevated levels of GABA+/water in the visual cortex of older adults. Several studies have reported GABA levels in other brain regions^30–32,37^, however, to the best of our knowledge, this is the first study to specifically compare visual cortical GABA concentration between older and younger adults. We additionally show associations between visual cortical GABA+/water levels and performance on two visual tasks that are hypothesised to, at least in part, be mediated by GABAergic inhibition. These associations were only present when participants of all ages were considered collectively. Increased GABA is specific to visual cortex with no alterations in GABA concentration in DLPFC. Furthermore, the observed associations between GABA levels and visual tasks were specific to visual cortex, with no significant correlation between visual performance and GABA levels in DLPFC.

While several perceptual studies have proposed an age-related decline in visual cortical GABAergic inhibition as a possible mechanistic explanation for behavioural findings^5,6,38^, there is a notable absence of data that directly addresses this hypothesis. There have been reports of reduced GABA levels in right hippocampus^30^, frontal^31^ and parietal^31^ cortices in older adults, and unaltered levels of GABA concentration in anterior cingulate cortex of older adults^30,31^, however visual cortical GABA data has not been reported. A gene expression approach (Western blot) has been used to investigate developmental changes to GABAergic system in post-mortem V1 tissues from humans aged between 20 days to 80 years^39^. No difference was found in protein expression of GAD65, GAD67, VGAT, GABAA ∝1, GABAA ∝2, and GABAA ∝3 between younger (n = 4, age 23–54 years) and older adults (n = 3, age 69–80 years), however the sample size was very small. Two out of 4 observers in their young adult group were above 50 years of age which is quite different from typical behavioural studies, where younger adults group usually include people between 18 to 30 years of age^6,16^). Nevertheless, visual inspection of the results of Pinto *et al*.^39^ shows a trend for increased expression of VGAT, GABAA ∝1, GABAA ∝3, and unaltered expression of GABAA ∝2 in older adults^39^, compared to younger adults. Previous reports of Glx concentration in the occipital^40^ and occipital-parietal lobe^41^ agree with our finding of decreased Glx in older adults.

The secondary questions explored in this study relate to whether measured GABA levels are associated with visual behavioural measures and the anatomical and neurochemical specificity of the age-related changes observed. We included binocular rivalry as a perceptual measure because of previous reports of a relationship between GABA concentration in visual cortex and rivalry parameters in other, non-elderly, human groups^26,27,42^. Consistent with previous findings^26,27^, we found longer percept durations to be associated with increased GABA levels within visual cortex when the entire participant group was considered, but not with visual cortical Glx levels or with GABA levels in DLPFC.

It is worth noting that the correlation between GABA levels and mean percept duration was not statistically significant when either age group was considered in isolation, which does not directly replicate the finding of van Loon *et al*.^26^ who identified a significant correlation between these indices (rho = 0.50 in 18 male adults of mean age 22 years). For our experiment, a-priori sample size estimation revealed that a sample of 28 participants would be needed to result in a power of 0.80 (type II error of 0.20) to detect a correlation of the magnitude reported in the Van Loon *et al*.^26^ study. Our sample size of 36 was chosen to test the hypothesis that the measures would be correlated across the whole data set (rather than in each age group independently). Indeed, our study was powered to find a correlation of at least 0.45 across the whole sample, but only of magnitude greater than 0.62 across each age group independently. There are several other key differences between our methods and those of Van Loon *et al*.^26^ namely, the specific stimuli used to elicit rivalry differed; and secondly, we included both men and women in our study.

While recognising that correlative evidence does not imply direct causality, our findings (and those of others) are consistent with a computational model of bistable perception in which perceptual switches are, at least partially, mediated via GABAergic inhibition between stimulus selective neural populations in visual cortex^26,27^. Our binocular rivalry data is consistent with previous studies demonstrating that healthy ageing results in stronger^43^ and prolonged interocular inhibition^20,21^. Increasing rivalry percept durations with healthy ageing suggest strengthened mutual inhibition between the conflicting percepts, and suggest a role for GABA in this inhibitory process. An alternate interpretation of our observed correlations is that they arise from independent ageing effects on GABA and binocular rivalry percept durations. Our data cannot rule out this explanation, however the neurochemical and anatomical specificity of our finding, along with the existence of a substantive body of previous literature indicating a role for GABAergic inhibition in binocular rivalry^26,27,42^, throws some doubt on this alternative explanation.

In our experimental design and participant instruction, we did not emphasise the collection of duration of mixed percept data (a period of perceptual mix of both red and green stimuli). A button to indicate mixed percept was available, however mixed percepts were only reported by a minority of participants (10 of 38: 5 older and 5 younger). The mixed percept condition may reflect weak interocular inhibition, and hence are of interest to models of inhibitory function. Unfortunately, we did not have sufficient mixed percept data to explore this issue, but expect it may be a fruitful area for future research.

As part of current attempts to understand age-related changes in visual function, spatial suppression of motion has received considerable attention^6,18,23^. A candidate mechanism previously proposed to explain behavioural observations is reduced neuronal spatial surround suppression, presumably due to diminished GABAergic functioning in older adults^6^. Consistent with previous literature, we found reduced spatial suppression in older adults^6,18,23^, however, in contrast to previous predictions, reduced perceptual surround suppression of motion was correlated with increased visual cortical GABA levels. We did not specifically measure GABA levels in hMT/V5, however, which is the visual brain area considered most relevant for motion perception tasks of this nature^35,36^, and which lies several centimetres away from the position of our occipital cortex voxel. While the possibility of highly localised regional differences in GABA levels across visual cortical regions seems unlikely (such that regionally reduced GABA in hMT/V5 occurs in the presence of increased GABA in V1) we have no direct evidence to either support or refute this possibility.

Alternately, if we assume that GABA levels in V1 may be reflective of those in hMT/V5, there are some recent neurophysiological results that may shed light on the direction of our observed correlation (reduced surround suppression in the presence of increased GABA). Recent work investigating the neural correlates of spatial suppression in alert behaving primates reports that measured spatial suppression of motion results from both surround suppression and correlated neural activity (i.e. noise correlations and signal correlation) in primate area MT/V5^44^. As stimulus size increases, the number of neurones generating the perceptual response also increases. Since the noise present in the activity of those neurones is correlated, the noise levels also increase with stimulus size and therefore perceptual responses are reduced for larger targets. GABA antagonists increase noise correlations in primate area MT/V5^45^, hence increased GABAergic inhibition should decrease correlated noise activity, leading to reduced spatial suppression of motion. Thus, our observation of reduced perceptual spatial suppression of motion being associated with increased visual cortical GABA is not completely at odds with contemporary neurophysiological understanding. Nevertheless, it is important to note that our correlative evidence can only hint at associations and further experiments are required to specifically address direct causative links.

We used MRS to quantify GABA concentration as it is a relatively standard tool that is applicable *in-vivo* in humans. MRS has limitations however for inferring mechanistic links between measured cortical metabolites and visual behaviour. Arguably the principal limitation is that MRS does not enable measurement of synaptic GABA activity, which is presumably more directly related to visual function than GABA concentration. Hence the exact mechanistic connection, if any, between our observed elevated GABA and visual behavioural alterations in older adults must remain somewhat speculative. Nevertheless, our principal finding of elevated GABA in older adults receives support from a recent gene expression study of older rhesus monkeys that reported upregulation of several components of the GABAergic system in primary visual cortex^46^. Furthermore, our behavioural findings are completely consistent with previous visual perceptual studies^6,18,20,21,23^, and there are plausible neurobiological explanations for our observed relationship between GABA levels and both visual tasks. The fact that a relationship between visual behaviour and GABA was only present in visual cortex and not DLPFC strengthens the likelihood that the observed correlations are of relevance to vision.

Here we used MRS with the MEGA PRESS sequence, currently the most commonly used and standard GABA estimation technique. Generally, GABA concentrations are calculated relative to unsuppressed water or total creatine. While the water signal has the advantage of increased SNR, total creatine has the advantage of being obtained in the same MEGA PRESS scan and has a resonance closer to GABA signal hence a similar chemical shift to GABA (for a review of MEGA PRESS current practices guidelines^47^, see Mullins *et al*.^47^). Our results show that both GABA+/water and GABA+/tCr levels are increased in the visual cortex of older adults, indicating that the observed differences between groups are not driven by the reference signal. GABA signals estimated using MEGA PRESS are contaminated by co-edited macromolecules^32^, however, the regional specificity of our findings (increased GABA in visual cortex but not DLPFC) makes it unlikely that our results are driven by an increase in macromolecules.

A further technical consideration important to the application of MRS to studies of adults of varying age is that healthy ageing has differential effects on tissue volume (see Supplementary material Figure S1). The amount of GABA varies within a voxel based on its tissue composition: grey matter contains approximately twice the GABA compared to white matter^48^, whereas cerebrospinal fluid is commonly assumed to contain negligible GABA. These issues complicate the measurement of GABA concentration within MRS voxels, which by necessity are required to be fairly large. In our analysis of GABA concentration in visual cortex, we compensated for anatomical differences in tissue structure between older and younger adults to ensure that these were not solely driving the observed between-group differences. In the absence of tissue composition correction, our raw data showed a trend towards increased GABA+/water (*t* (1,36) = −1.64, P = 0.11) and GABA+/Creatine (*t* (1,36) = −2.01, P = 0.05) in older adults relative to younger adults. Raw GABA+/Choline values were also significantly higher in the older group (*t* (1,36) = −3.12, P < 0.01) (see Supplementary Figure S3). Our chosen method of tissue compensation correction^49^ is specifically designed to remove the dependency of GABA+/water values on tissue fraction and is currently considered to be the most suitable to apply to conditions where there is brain atrophy.

In summary, via neuroimaging and perceptual experiments in the same observers, we demonstrate increased GABA+/water levels in the visual cortex of older adults. We show that measured GABA+/water levels in visual cortex are associated with visual performance on two separate tasks when adults of varying age are included within the sample, and that this association is not present in a non-visual brain region. Our findings challenge prior assumptions regarding age related changes to GABAergic inhibition within the visual system, and suggest that previous interpretations regarding the underlying mechanisms driving certain age-related changes to suppressive visual perceptual phenomena require rethought and further experimentation.

## Methods

### Experimental Design and Statistical Analysis

An observational case-control design was used. Twenty younger (11 males: aged 20–34 years) and 20 older adults (6 males: aged 63–78 years) participated in the study and were recruited as a convenience sample from a database of previous participants and via advertisements placed at the University of Melbourne and Monash University. All participants had best corrected visual acuity of 6/7.5 or better and were refractively corrected for the working distance. Participants underwent a screening optometric examination to exclude for any ocular disease. The study was approved by the Monash University Human Research Ethics Committee and all procedures adhered to the Declaration of Helsinki. Written informed consent was provided by all participants.

The sample size was determined a-priori based on the following convergence of estimates (all for alpha = 0.05, beta = 0.2): a) data^31^ from Gao *et al*. (2013), to estimate expected group difference and variance in GABA levels between older and younger groups resulted in a required sample of 10 in each group, b) data^21^ from Ukai *et al*. (2003) to estimate expected difference and variance in binocular rivalry rates between older and younger groups resulted in a required sample of 18 in each group; c) data^6^ from Betts *et al*. (2005) for the expected difference and variance in motion suppression index between older and younger groups resulted in required sample of 12 in each group; and d) data^26^ from van Loon *et al*. (2013) estimated an expected correlation between median percept duration and GABA levels of r = 0.45, leading to a sample size estimate of a total of 36 individuals (across groups). Hence, we recruited 20 into each group, with the aim of collecting a minimum of a 36 completed datasets for each task.

All statistical tests were computed using IBM SPSS Statistics 20 (SPSS Inc., Chicago, IL). All data sets were tested for normality using a Kolmogorov–Smirnov test. Group data were compared using t-tests and correlations were performed using Pearson correlational analysis.

### Magnetic Resonance Spectroscopy (MRS)

A 3 T MRI scanner (MAGNETOM Skyra, Siemens, Erlangen, Germany) with 32 channel headcoil was used to collect the T1 weighted whole brain image (MPRAGE, TR/TE 58 msec/3.7 ms, 1 mm^3^ isotropic voxels) and single voxel spectroscopy data with the following parameters: repetition time [TR] = 1500 ms; echo time [TE] = 68 ms; voxel size = 30 × 25 × 20 mm^3^). All participants watched the same animated movie while in the scanner. A prototype GABA-specific sequence of Point Resolved Spectroscopy (PRESS) with a previously described MEGA suppression scheme was used to acquire the ^1^H J-difference spectra^33^. A total of 192 transients (which consist of two TRs) were acquired over a period of 9 minutes. During the odd transients, a frequency selective inversion pulse known as an editing pulse was applied to the ^3^CH_2_ resonance (1.9 ppm) of GABA (EDIT ON). During the even transients, the editing pulse was applied at 7.46 ppm (EDIT OFF). The difference between the EDIT ON and EDIT OFF sub-spectra was used to quantify GABA concentration. The unsuppressed water signal (8 averages) was acquired from the same voxel. The visual cortex voxel was placed on either side of the calcarine sulcus 6 mm anterior to the dura. Voxel positions were carefully chosen to avoid the meninges, large blood vessels and ventricles. The GABA concentration relative to water levels was quantified from the edited difference spectrum using Gannet software^25^ (example spectra are shown in Fig. 1A–D). Since the edited GABA signal is contaminated by co-edited macromolecules^32^, GABA values are referred to as GABA+ in this paper. GABA fit errors were used to assess the quality of spectra. All of the spectra had a GABA fit error < 10%. The fit quality metrics (mean ± SD: Cr resonance FWHM: 8.01 ± 0.54; GABA resonance FWHM: 18.16 ± 1.05; Cr fit error: 10.46 ± 1.29; GABA fit error: 6.24 ± 0.99; GABA signal-to-noise ratio: 16.41 ± 2.63) were comparable to previous studies^50,51^ (for individual and group fit quality metrics, see Supplementary Material Table S1).

Ageing causes cortical atrophy, which changes the brain composition within our voxel of interest. Harris *et al*. (2015) proposed a comprehensive tissue composition correction resulting in GABA+ values that are minimally dependent on grey/white matter fractions in simulated voxels with different amounts of brain atrophy^49^. Accordingly, we segmented the T1 weighted 3D images into grey matter (${GM}$), white matter (${WM}$) and cerebrospinal fluid (${CSF}$) using SPM8 software^52^, and corrected our GABA+ values using the following equation^49^:

2 $${C}_{fullcorr}=\frac{MM{I}_{G}}{k{I}_{W}}(\frac{{\sum }_{i}^{GM,WM,CSF}{c}_{w,i}\exp (-\frac{TE}{{T}_{2W,i}})(1-\exp (-\frac{TR}{{T}_{1W,i}}))\,{f}_{i}}{\exp (-\frac{TE}{{T}_{2G}})(1-\exp (-\frac{TR}{{T}_{1G}}))})(\frac{{\mu }_{GM}+\alpha {\mu }_{WM}}{(\,{f}_{GM}+\alpha {f}_{WM})({\mu }_{GM}+\alpha {\mu }_{WM})})$$

where *MM* is the correction factor for the co-edited macromolecular signal, *I*_*G*_ and *I*_*w*_ are the GABA and water signal integrals, *C*_*w*_ is the visible water concentration, k is the editing efficiency of GABA, *T*_1*G*_ *T*_1*w*_ *T*_2*G*_ *T*_2*w*_ are the T1 and T2 relaxation time constants for GABA and water, *TE* is echo time, *TR* is repetition time, *f*_*GM*_ and *f*_*WM*_ are the grey matter and white matter volume fractions, μ_*GM*_ and μ_*WM*_ are the group average grey matter and white matter fractions from our younger group (as suggested by Harris *et al*.^49^), and ∝ is the assumed ratio between GABA concentrations in grey matter and white matter. The equation and the values for *MM*, *k*, ${C}_{w},\,{T}_{1G},\,\,{T}_{1w},{T}_{2G},{T}_{2w}$, and ∝ were taken from the GannetQuantify routine from the Gannet toolbox^25^.

As this study was primarily aimed at identifying GABA related changes in the ageing cortex, we used the MEGA PRESS sequence which was optimised for the GABA signal. However, from the same spectrum it was also possible to estimate the levels of glutamate, the main excitatory neurotransmitter in the brain, albeit with slightly less precision. We quantified edited Glx, a combined estimate of glutamate and glutamine, using Gannet software^25^. Edited Glx values were normalised to water and corrected for partial volume differences using the following equation:

3 $$Gl{x}_{corr}=Glx\times \frac{1}{1-{f}_{CSF}}.$$

where *Glx*_*corr*_ is the cerebrospinal fluid fraction corrected edited Glx/water, *Glx* is the raw Glx/water value in arbitrary units, and *f*_*CSF*_ is the voxel cerebrospinal fluid fraction.

Our main region of interest for MRS was visual cortex, however, we additionally collected data from (DLPFC). MRS is a rapidly advancing field, and our data collection began before currently considered optimal methods for tissue correction were published^49^. Our planned analysis and acquisition techniques were instead modelled on a previous study that reported an association between GABA levels and binocular rivalry^26^. For this reason, we did not collect unsuppressed water data in DLPFC since our original intention was to normalise GABA+ to total creatine (tCr) for all hypothesis testing. Normalisation to total creatine (tCr), is just as, or more, reproducible as referencing to water^53^, however the water referencing has some advantages for tissue composition correction^49^. We additionally completed the analysis of visual cortex data using GABA+/tCr levels to enable regional comparison. In both areas, GABA+/tCr levels were corrected for CSF fraction, as the concentration of GABA and creatine is commonly considered to be negligible in CSF^31,54,55^. The methodological aspects of this analysis including referencing to tCr, voxel size, and voxel position were identical to a key previous study that motivated our work^26^ and were replicated to enable direct comparison.

### Behavioural testing experimental setup

The behavioural testing was conducted in a dark room under binocular viewing. The viewing distance was 100 cm and 57 cm for spatial suppression of motion and binocular rivalry, respectively. A chinrest was used to stabilise head position. Stimuli were displayed on a gamma-corrected Sony G520 21-inch CRT monitor (refresh rate: 120 Hz, display resolution: 800 × 600 pixels, maximum luminance: 100 cd/m^2^) via a VSG ViSaGe graphics system (Cambridge Research Systems, Kent, UK). The software for stimulus generation and threshold estimation were written in Matlab™ version 7 (The MathWorks.inc., USA) and VSG toolbox (Cambridge Research Systems, Kent, UK).

### Binocular rivalry experimental procedure

**Figure 5:**
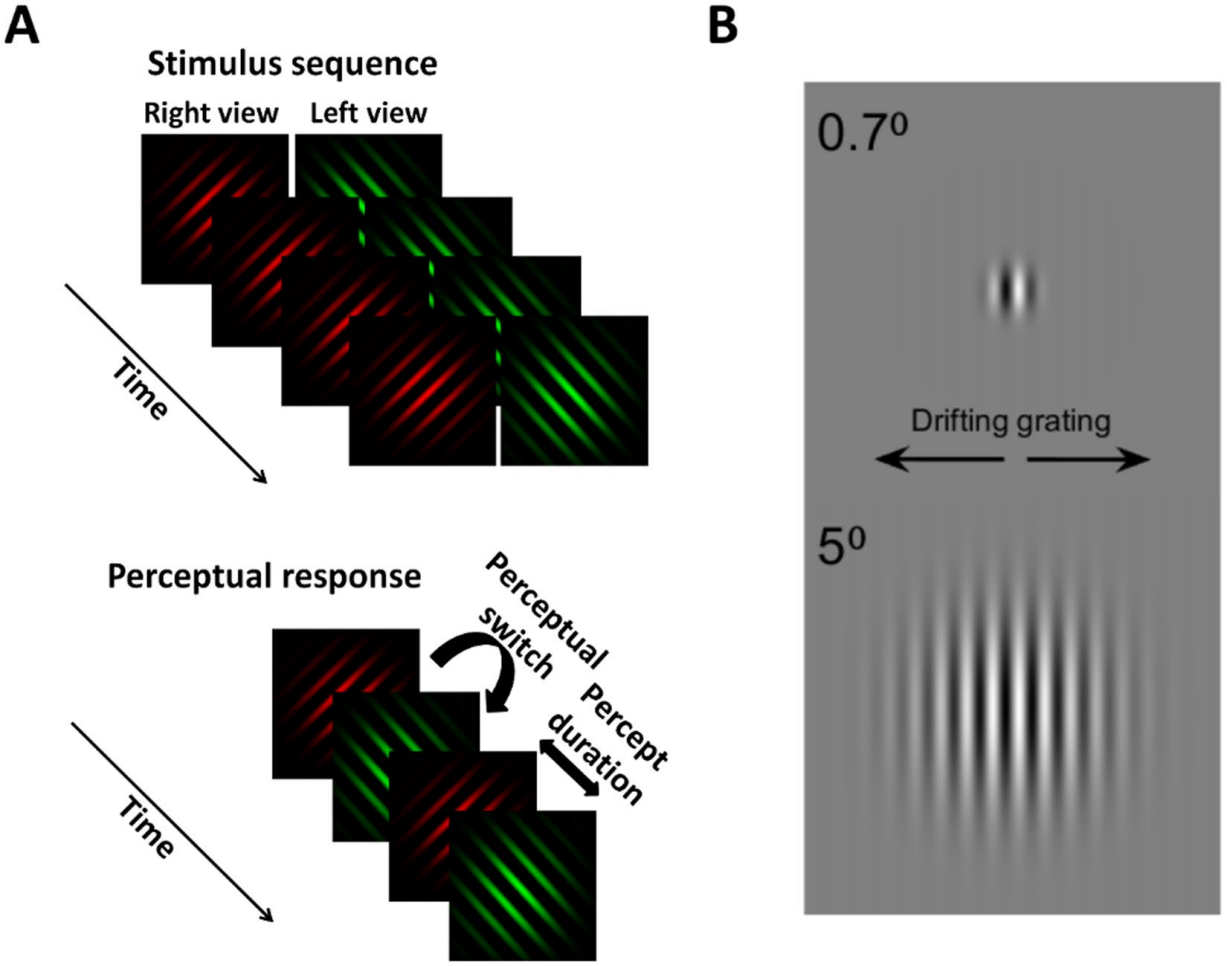
(**A**) Stimulus sequence used to assess binocular rivalry. (**B**) Example stimuli used to assess spatial suppression of motion.

Within one hour of the MRS procedure, we measured performance on two visual tasks involving suppression. The tests were performed in random order. For the binocular rivalry task, we used circularly windowed sine gratings (100% contrast, 4° diameter, 1.5 c/deg spatial frequency), which were viewed through a mirror stereoscope (right eye: red grating, oriented 45°, left eye: green grating, oriented 135°). Participants were asked to report what they saw i.e. red percept or green percept by button press (Fig. 5A). Participants were given the option to press a 3rd button if they experienced a mixed percept; however, no explicit instructions regarding the criteria for judging mixed percepts were provided. Stimuli were displayed for 90 seconds and repeated four times. Mean percept durations and switch rate for each repeat were calculated and averaged. One younger and one older participant could not do the rivalry task due to poor binocular fusion. All of the remaining participants were able to perform the task reliably. Mixed percepts were excluded for percept duration calculations. Ten out of 38 (26%) participants (5 older, 5 younger) reported mixed percepts; hence there was insufficient data to perform specific analyses relating mixed percept duration or frequency to our MRS measures.

### Spatial suppression of motion experimental procedure

For the spatial suppression of motion task, participants were required to identify the direction of motion of large, high-contrast (92%) drifting Gabor patterns. The targets were vertically oriented Gabor stimuli of 92% contrast, 1 c/deg spatial frequency, and drifting at a speed of 2°/sec. Consistent with previous studies^6,35^, the Gabor targets were 0.7° and 5° of visual angle, where the size is defined as 2σ of the Gaussian envelope (see Fig. 5B).

To determine the stimulus duration required to correctly discriminate direction of motion, participants were asked to indicate the direction of motion of each stimulus presentation (right or left) by a button press. Duration thresholds were obtained using two interleaved adaptive staircases (3-down 1-up) that yielded threshold at approximately the 79.4% correct response level on a psychometric function^56^. Each staircase was terminated after four reversals. The last two reversals of each staircase were averaged to determine the threshold, with the final threshold estimate being determined as the average of the two staircase outcomes. Suppression indices were calculated using equation (1).

### Numbers of participants reported in the results

We did not obtain an unsuppressed water signal in visual cortex from one younger participant, and spectroscopy data from one younger participant was discarded due to excessive frequency drift (see Supplementary Figure 1). Hence the reported spectroscopy data are from 18 younger and 20 older adults, data for the motion suppression task are from 20 younger and 20 older adults, and data for the binocular rivalry task are from 19 younger and 19 older adults. The datasets consisting raw spectra and behavioural data of the current study are available from the corresponding author on reasonable request.

## Electronic supplementary material

Supplementary material
