# Supplementary material for: Occipital GABA levels in older adults and their relationship to visual perceptual suppression
Source: Sci Rep. 2017 Oct 27;7:14231. doi: 10.1038/s41598-017-14577-5 (PMC5660206; doi:10.1038/s41598-017-14577-5)
Supplement: Supplementary file 1 — Supplementary material [file 41598_2017_14577_MOESM1_ESM.doc]

**Title**: Occipital GABA levels in older adults and their relationship to visual perceptual suppression

**Authors**

**Kabilan Pitchaimuthu** (Department of Optometry and Vision Sciences, University of Melbourne, Parkville VIC 3010, Australia)

**Qi-zhu Wu** (Monash Biomedical Imaging, Monash University, Clayton VIC 3800, Australia)

**Olivia Carter** (Melbourne School of Psychological Sciences, University of Melbourne, Parkville VIC 3010, Australia)

**Bao N Nguyen** (Department of Optometry and Vision Sciences, University of Melbourne, Parkville VIC 3010, Australia)

**Sinyeob Ahn** (Magnetic Resonance, Siemens Medical Solutions USA Inc, San Francisco, CA 94116, USA)

**Gary F Egan** (Monash Biomedical Imaging, Monash University, Clayton VIC 3800, Australia)

***Allison M McKendrick** (Department of Optometry and Vision Sciences, University of Melbourne, Parkville VIC 3010, Australia)


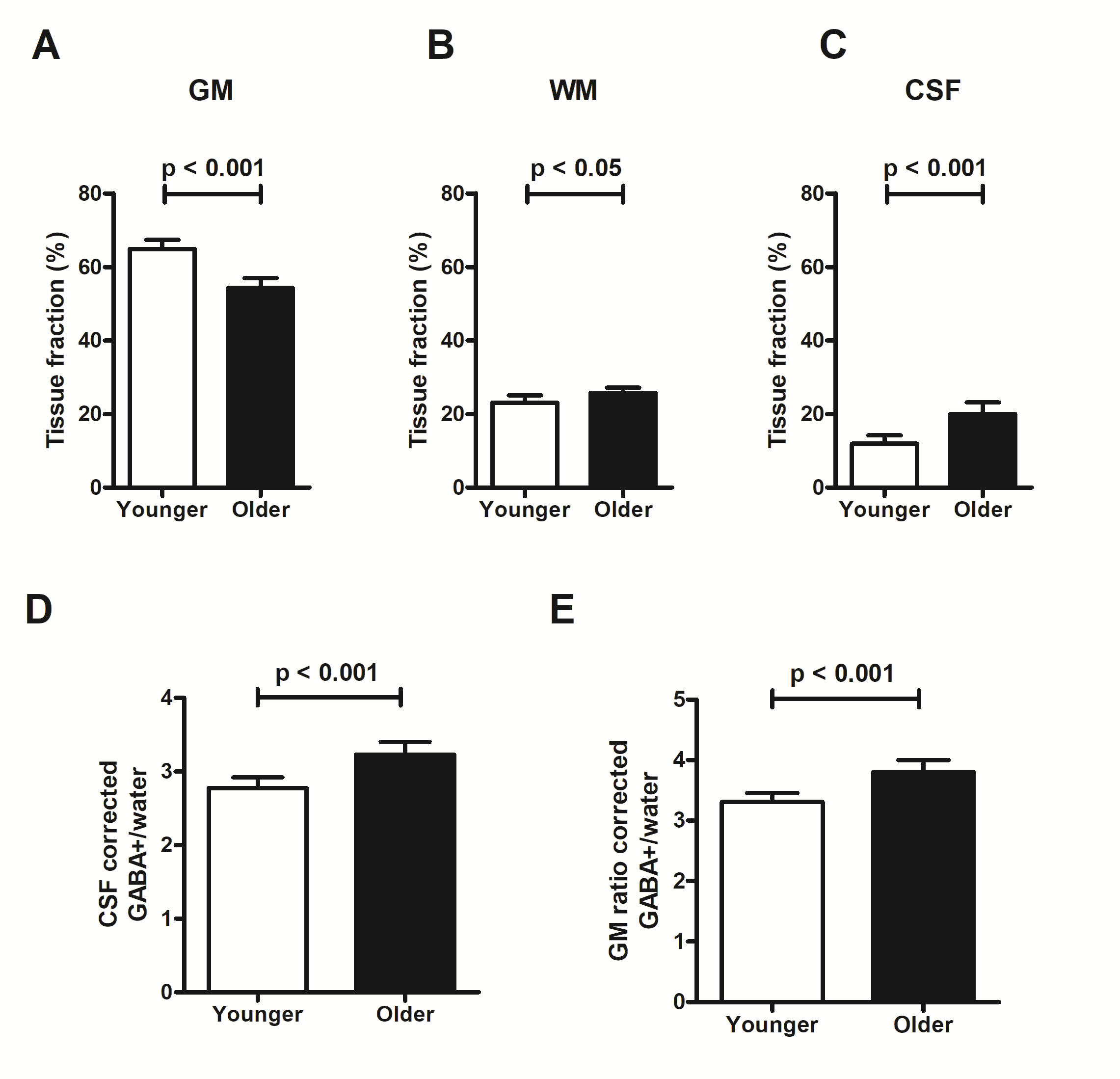


***Supplementary Figure S1****. Average group (A) white matter (WM) tissue fraction (B) grey matter (GM) tissue fraction (C) cerebrospinal fluid (CSF) tissue fraction (D) CSF-corrected GABA+/water levels, and (E) GM ratio corrected GABA+/water levels. Error bars are 95% confidence intervals of the mean.*

**
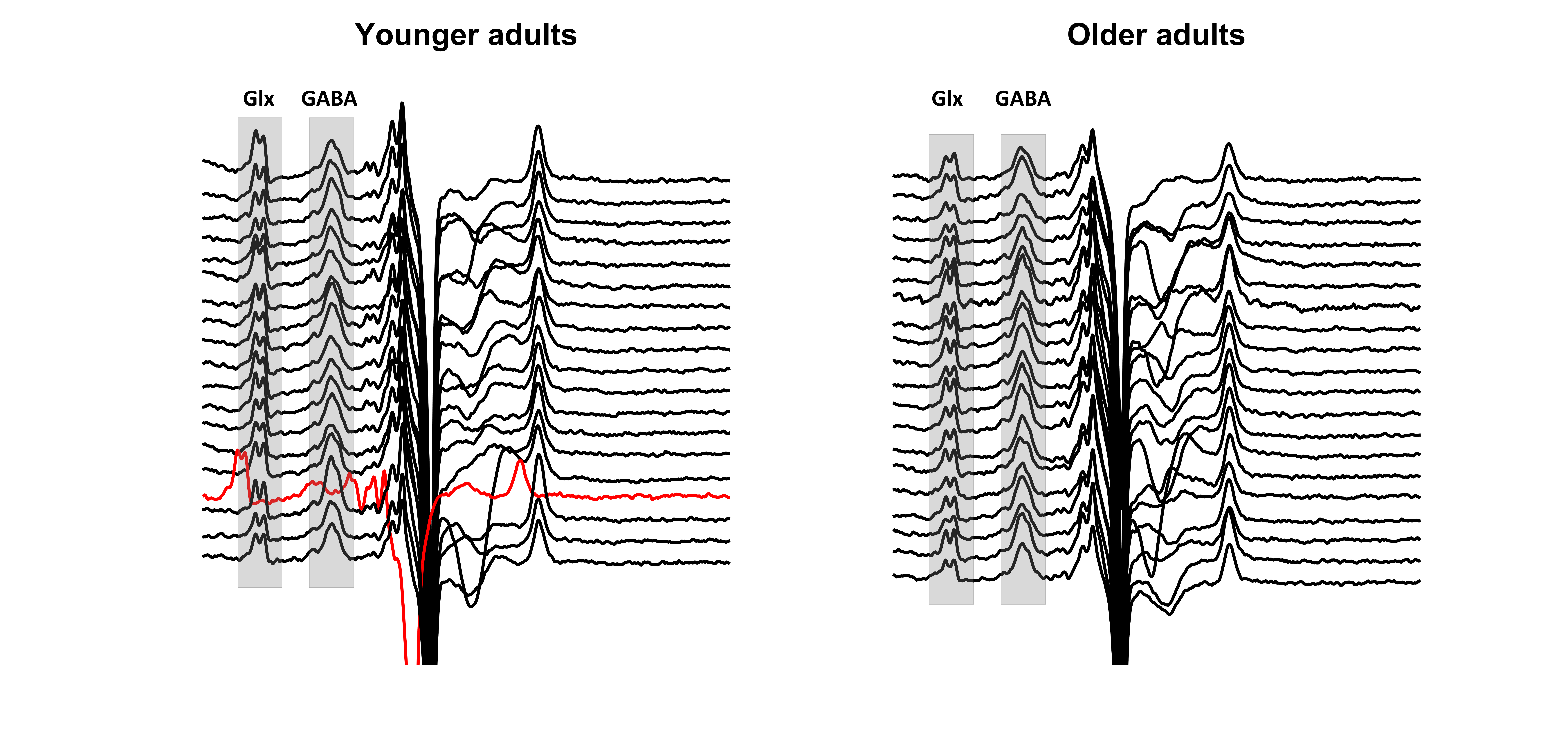
*****Supplementary Figure S2.*** *Raw spectra from all younger and older participants. The spectrum from one younger participant was discarded due to excessive frequency drift, which is indicated in red colour.*

**
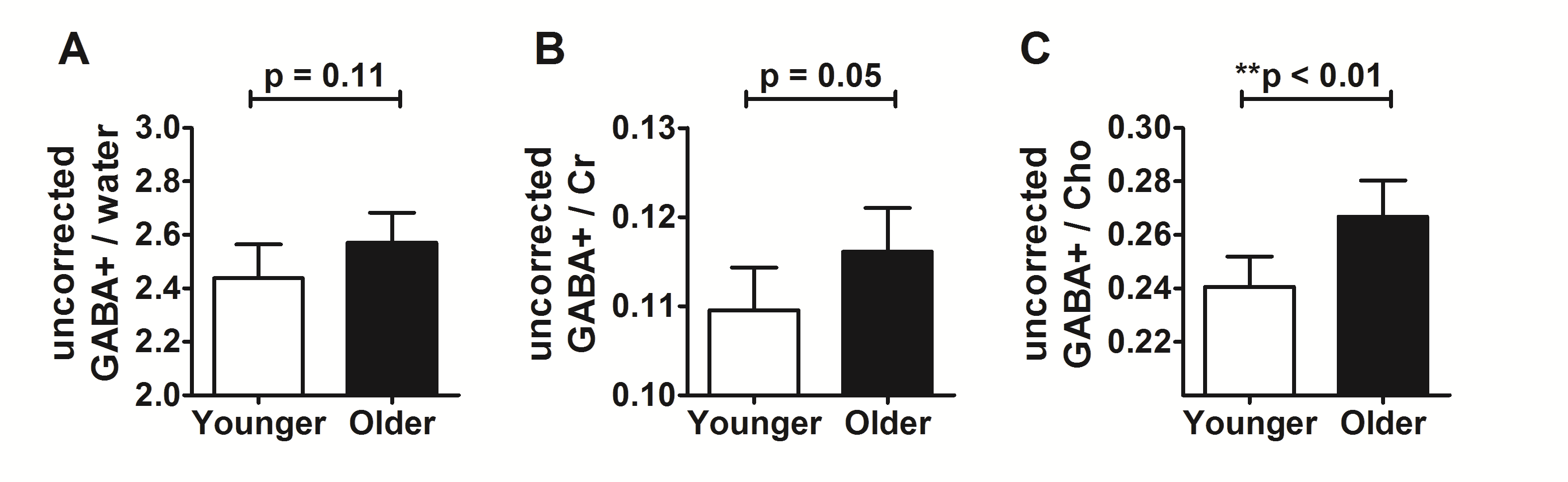
**

***Supplementary Figure S3.*** *Tissue uncorrected visual cortical GABA+ concentration values normalised to water (A), creatine (B), and choline (C). There is a trend towards increased GABA+/water and GABA+/Cr levels, and a statistically significant increase in GABA+/Cho levels in older adults. Error bars are 95% confidence intervals of the mean.*

***Supplementary Table S1.*** Individual fit quality metrics for quantification of GABA and Glx in visual cortex. Group differences in each of the fit quality metrics are given at bottom of table. FE = fit error, FWHM = full width at half maximum, SNR = signal to noise ratio.

| Subject number | Age group | Visual cortical GABA | | | Visual cortical Glx | | |
| --- | --- | --- | --- | --- | --- | --- | --- |
|  |  | FE | FWHM | SNR | FE | FWHM | SNR |
| 1 | Younger | 5.7383 | 18.378 | 17.427 | 6.2736 | 15.718 | 15.94 |
| 2 | Younger | 6.659 | 19.002 | 15.017 | 5.3551 | 14.051 | 18.674 |
| 3 | Younger | 6.6106 | 16.914 | 15.127 | 6.1105 | 12.958 | 16.365 |
| 4 | Younger | 6.5846 | 16.655 | 15.187 | 6.3625 | 14.032 | 15.717 |
| 5 | Younger | 7.1439 | 18.165 | 13.998 | 7.3073 | 16.272 | 13.685 |
| 6 | Younger | 7.6468 | 19.998 | 13.077 | 5.4535 | 15.658 | 18.337 |
| 7 | Younger | 6.5324 | 17.118 | 15.308 | 6.2459 | 14.058 | 16.01 |
| 8 | Younger | 6.674 | 17.775 | 14.984 | 6.1333 | 15.758 | 16.304 |
| 9 | Younger | 7.4863 | 18.631 | 13.358 | 4.5168 | 17.084 | 22.139 |
| 10 | Younger | 6.3434 | 19.315 | 15.764 | 6.2975 | 14.868 | 15.879 |
| 11 | Younger | 8.0549 | 19.525 | 12.415 | 6.4353 | 16.64 | 15.539 |
| 12 | Younger | 7.2966 | 17.182 | 13.705 | 6.9847 | 15.46 | 14.317 |
| 13 | Younger | 6.2527 | 18.496 | 15.993 | 5.7797 | 15.55 | 17.302 |
| 14 | Younger | 6.1526 | 19.312 | 16.253 | 5.1065 | 16.468 | 19.583 |
| 15 | Younger | 6.1103 | 18.276 | 16.366 | 4.5988 | 14.34 | 21.745 |
| 16 | Younger | 5.9057 | 16.813 | 16.933 | 5.8822 | 12.481 | 17 |
| 17 | Younger | 7.4155 | 18.321 | 13.485 | 5.8476 | 14.75 | 17.101 |
| 18 | Younger | 8.1878 | 17.957 | 12.213 | 5.5025 | 15.475 | 18.174 |
| 19 | Older | 4.7311 | 20.218 | 21.137 | 4.6998 | 14.584 | 21.277 |
| 20 | Older | 5.498 | 18.931 | 18.188 | 6.209 | 18.192 | 16.106 |
| 21 | Older | 8.3288 | 17.861 | 12.006 | 6.6474 | 15.621 | 15.043 |
| 22 | Older | 4.5062 | 20.647 | 22.192 | 5.3132 | 13.544 | 18.821 |
| 23 | Older | 6.8621 | 16.629 | 14.573 | 5.6062 | 14.994 | 17.838 |
| 24 | Older | 4.7958 | 16.957 | 20.852 | 7.0958 | 16.218 | 14.093 |
| 25 | Older | 5.2358 | 19.179 | 19.099 | 6.0903 | 14.719 | 16.42 |
| 26 | Older | 6.0497 | 17.274 | 16.53 | 5.7868 | 13.705 | 17.281 |
| 27 | Older | 6.309 | 18.414 | 15.85 | 4.9838 | 14.031 | 20.065 |
| 28 | Older | 5.8648 | 18.722 | 17.051 | 5.5718 | 14.568 | 17.948 |
| 29 | Older | 5.7177 | 16.498 | 17.49 | 3.7924 | 15.065 | 26.368 |
| 30 | Older | 6.4865 | 18.069 | 15.417 | 5.7676 | 13.922 | 17.338 |
| 31 | Older | 6.3008 | 17.493 | 15.871 | 6.0921 | 16.66 | 16.415 |
| 32 | Older | 5.026 | 17.982 | 19.896 | 6.1307 | 15.462 | 16.311 |
| 33 | Older | 5.9447 | 18.684 | 16.822 | 5.2983 | 14.903 | 18.874 |
| 34 | Older | 5.4285 | 16.684 | 18.421 | 8.5471 | 17.809 | 11.7 |
| 35 | Older | 5.8525 | 17.778 | 17.087 | 7.79 | 16.139 | 12.837 |
| 36 | Older | 5.9828 | 18.817 | 16.715 | 4.3392 | 17.14 | 23.046 |
| 37 | Older | 4.6177 | 17.689 | 21.656 | 4.8034 | 13.305 | 20.818 |
| 38 | Older | 4.9715 | 17.894 | 20.115 | 8.999 | 21.09 | 11.112 |
|  | Group comparison | t(36)=4.09, p=0.0002 | t(36)=0.27, p=0.79 | t(36)=4.32, p=0.0001 | t(36)=0.22, p=0.83 | t(36)=0.94, p=0.36 | t(36)=0.27, p=0.79 |
